# Bispecific antibodies in immunotherapy for acute leukemia: latest updates from the 66th annual meeting of the American society of hematology, 2024

**DOI:** 10.3389/fonc.2025.1566202

**Published:** 2025-04-09

**Authors:** Weijie Cao, Shuhan Ma, Lijie Han, Hanzhou Xing, Yingmei Li, Zhongxing Jiang, Xuejun Guo, Jifeng Yu

**Affiliations:** ^1^ Department of Hematology, The First Affiliated Hospital of Zhengzhou University, Zhengzhou, Henan, China; ^2^ Department of Hematology/Oncology, Puyang Oilfield General Hospital Affiliated with Xinxiang Medical College, Puyang, China

**Keywords:** bispecific antibodies, acute lymphoblastic leukemia, acute myeloid leukemia, myelodysplastic syndrome (MDS), immunotherapy

## Abstract

Bispecific antibodies (BsAbs) are cutting-edge immunotherapy agents that can bind two distinct antigens or epitopes simultaneously. They hold significant potential in targeting leukemic cell markers and activating immune cells like T cells or NK cells to eliminate malignant cells. BsAb treatments showed encouraging outcomes for both acute myeloid leukemia (AML) and acute lymphoblastic leukemia (ALL). In relapsed/refractory (R/R) ALL, BsAbs improved overall survival (OS) and achieved measurable residual disease (MRD) negativity in most patients. Blinatumomab plus standard chemotherapy or in combination with other treatments, such as Mini-Hyper-CVD and Inotuzumab Ozogamicin, improved disease-free survival (DFS) in B-ALL. In AML and related conditions, novel BsAbs like AFM28 (CD123xCD16A) and Vibecotamab (CD123xCD3) showed promising efficacy in heavily pretreated R/R AML and in MDS/CMML following the failure of treatment with hypomethylating agents (HMA). The meeting underscored the transformative potential of BsAbs, especially in ALL-focused trials, with ongoing research aiming to evaluate their safety and efficacy in broader patient populations and combination regimens. This summary highlights the latest progress in BsAb-based immunotherapy presented at the ASH 2024 meeting, held from December 7–10 in San Diego, California.

## Introduction

Bispecific antibodies (BsAbs) offer a novel and promising approach in cancer immunotherapy. With two distinct binding domains, these antibodies can simultaneously target either two different antigens or two epitopes of a single antigen. Recently, various BsAbs, such as CD19xCD3, CD123xCD16A, and CD123xCD3, have been developed to target specific B-cell markers or myeloid-cell markers on malignant leukemic cells with the goal of eradicating leukemic cells by engaging T cells or NK cells. These BsAbs have previously shown encouraging outcomes in heavily pretreated patients with relapsed/refractory (R/R) acute lymphoblastic leukemia (ALL) and acute myeloid leukemia (AML) (1, 2). This summary highlights the latest advancements in BsAb-based immunotherapy for acute leukemia, as presented at the 66th American Society of Hematology (ASH) 2024 annual meeting, held from December 7–10 in San Diego, California. Using the words “antibody” and “acute leukemia”, “Bispecific” and “acute leukemia” combination search, we found 161 and 78 abstracts, respectively. We have selected 9 representative abstracts from these abstracts to summarize the novel bispecific antibodies and bispecific T-cell engagers (BITEs) that have entered clinical trials for the treatment of acute leukemia.

## BsAb immunotherapy in ALL

Blinatumomab, a bispecific antibody (BsAb), helps CD3-positive T cells recognize and eliminate CD19-positive ALL. It has been approved for use in patients with R/R ALL. Research has shown that blinatumomab treatment significantly improves overall survival (OS) compared to chemotherapy in R/R B-ALL patients. Additionally, it has proven to be both safe and effective as a first-line therapy for children and young adults with B-ALL who are either resistant or intolerant to chemotherapy (1, 2). Numerous clinical trials are also underway to assess its use in R/R B-ALL, particularly in Philadelphia chromosome-positive (Ph+) ALL (**Table 1**).

**Table 1:** Clinical trials investigating bispecific antibodies for patients with acute leukemia.

ASH Abstract# (Reference #)	Leukemia types	Clinical trial phase	Regimen	Setting	Enroll number	Median number of prior LOT (range)	Median age (range) years	mFU (range) month	CR% or mCR%	ORR%	mDOR, month (range)	mPFS,or OS month	CRS total incidence % (grade >3, %)
1440 (#^3^)	ALL	Ib	SC blinatumomab as monotherapy	R/R B-ALL	27	2 (1-5)	52 (19-78)	5.0 (0.49-10.9)	89%	89%	5.2 (0.66-10.9)	3.8 (1.8-8.4)	N/A
2811 (#^4^)		II	Mini-Hyper-CVD with Inotuzumab Ozogamicin and Blinatumomab	R/R B-ALL	133	NA	37 (17-87)	40 (3-136)	65.00%	86.00%	N/A	3 yrs OS 45%, RFS 44%	N/A
1431.1 (#^5^)		II	Hyper-CVAD, with or without Inotuzumab Ozogamicin, and Sequential Blinatumomab	Newly diagnosed B-ALL	75	N/A	33 (18-59)	38 (5-91)	100.00%	100.00%	N/A	3 yrs OS 90%	N/A
1439 (#^6^)		II	Combination of 3rd Generation TKI Olverembatinib, Blinatumomab and Chidamide	Older newly diagnosed Ph+ ALL	9	N/A	61 (50-73)	14	100%	100%	N/A	1.5 yrs OS and EFS 100%	11% (0%)
1 (#^8^)		III	Blinatumomab Added to Chemotherapy	Newly diagnosed standard risk children B-ALL	1440	N/A	4.3 (2.8-6.4)	2.5 yrs(1.6-3.2 yrs)	N/A	N/A	N/A	3 yrs DFS 96.0% in treatment group vs 87.9% in control group; 3 yrs DFS 97.5% in treatment group vs 84.8% in control group.	N/A (0.3%)
835 (#^9^)		III	Frontline Ponatinib Plus Blinatumomab	Adult Ph+ ALL Patients of All Ages	133	N/A	57 (20-87)	6.4 (0.1-32.3)	98%	98%	N/A	Estimated 18-months OS 91.6%	N/A (1.5%)
738 (#^10^)	AML	I	bispecific CD123/CD16A ICE	R/R AML	24	2 (1-3)	72 (28-82)	N/A	33% CR in 250mg group; 50% CR in 300mg group	N/A	N/A	N/A	8.3% (4.2%)
1007 (#^11^)		II	Vibecotamab, a CD123xCD3 Bispecific T-Cell Engaging Antibody	MDS or CMML after Hypomethylating Failure and in MRD-Positive AML	37	N/A	N/A	N/A	19 MDS, respond rate 68%,mCR 63%; 18 AML, 28% MRD-	N/A	N/A	N/A	68% (5%)

BsAbs, bispecific antibodies; R/R B-ALL, relapsed/refractory B-cell acute lymphoblastic leukemia; T-ALL, T-cell acute lymphoblastic leukemia; R/R AML, relapsed/refractory acute myeloid leukemia; NR, not reached; NA, not available; CR, complete response; mCR, marrow complete remission; CRS, cytokine release syndrome; LOT, lines of therapy; mDOR, median duration of response; mPFS, median progression free survival; OS, overall survival; mFU, median follow-up; CMR, complete molecular response; ORR, overall response rate; mORR, median overall response rate; CI, confidence interval; CRi, CR with incomplete hematologic recovery; MDS, myelodysplastic syndrome; CMML, chronic myelomonocytic leukemia; MRD, minimal residual disease.

A phase 1b trial with long-term follow-up found that subcutaneous (SC) blinatumomab treatment in heavily pretreated patients with R/R B-ALL resulted in high response rates and sustained remissions. Among the 27 patients, 24 (89%) achieved complete remission (CR) or CR with partial/incomplete hematological recovery (CRh/CRi) within two treatment cycles. In the 250μg/500μg dose group, 86% reached remission, while 92% in the 500μg/1000μg group did the same. In terms of measurable residual disease (MRD), 83% and 100% of responders in the two respective groups were MRD-negative (MRD <10^-4^). After a median follow-up of 5.0 months (range 0.49–10.9), 88% of patients remained relapse-free, with a median overall survival (OS) of 9.8 months (range 6.5–14.3 months) (3).

A phase II trial investigating the combination of Mini-Hyper-CVD, Inotuzumab Ozogamicin (INO), and blinatumomab in R/R B-ALL patients showed promising results. The overall response rate (ORR) was 86%, with 65% achieving CR, among the 132 evaluable patients. Assessed by flow cytometry, MRD negativity was observed in 53% of patients following the first treatment cycle and in 85% overall. Following a median follow-up of 40 months (range 3–136), the 3-year OS and relapse-free survival (RFS) rates were 45% and 44%, respectively. The “dose-dense” (D-D) regimen, which involved administering Mini-Hyper-CVD and INO along with blinatumomab from day 4 to day 21 in a 28-day cycle for up to 6 cycles, yielded significantly improved outcomes. The 1-year OS rate for the D-D regimen was 94%, compared to 51% in Cohort 1 (Mini-Hyper-CVD and INO) and 66% in Cohort 2 (Mini-Hyper-CVD, INO, and blinatumomab for 4 cycles). The combination of blinatumomab and fractionated INO improved both safety and efficacy. The D-D approach showed high rates of early and deep MRD responses, suggesting it could be more effective than sequential treatment with these agents (4). A phase II trial combining Mini-Hyper-CVD, rituximab, INO, and blinatumomab in pediatric R/R B-ALL is also ongoing, with results pending (5).

A phase II study of Hyper-CVAD, with or without INO, and sequential blinatumomab in newly diagnosed B-ALL patients demonstrated that adding INO to the Hyper-CVAD + blinatumomab regimen improved overall OS in 75 patients with Ph-negative B-ALL. With a median follow-up of 38 months (range, 5–91 months), the 30-month RFS rates were 91% in the INO group versus 74% in the non-INO group (P=0.05), and OS rates were 100% versus 82% (P=0.008). In high-risk patients, the 30-month RFS was 92% in the INO group compared to 67% in the non-INO group (P=0.07), with OS rates of 100% versus 76% (P=0.05) (6).

A study evaluating the combination of Olverembatinib, Blinatumomab, and Chidamide (ABC regimen) in older patients with newly diagnosed Ph+ ALL demonstrated strong efficacy and safety. Among 9 patients, the regimen achieved an 88.8% complete molecular response (CMR) rate at 3 months. Additionally, the 1.5-year OS and event-free survival (EFS) rates were both 100%, with no relapses or deaths observed. These promising results suggest that the ABC regimen may significantly improve long-term survival in this patient population (7).

A phase III trial demonstrated that adding blinatumomab to chemotherapy improves DFS in newly diagnosed pediatric B-ALL with standard-risk. With a median follow-up of 2.5 years (IQR = 1.6-3.2) and 1440 evaluable patients, the 3-year DFS was 96.0 ± 1.2% for those in the blinatumomab group, compared to 87.9 ± 2.1% in the control group. This addition represents a significant breakthrough, establishing a new standard of care with important implications for treating children with newly diagnosed B-ALL (8).

Another phase III trial evaluating frontline ponatinib plus blinatumomab in adult Ph+ ALL patients showed promising results. Among 95 evaluable patients, 93 (98%) achieved complete hematologic remission (CHR), and 73% had a MRD response, including CMR and positive non-quantifiable results. After a median follow-up of 6.4 months (range 0.1–32.3), the estimated 18-month OS rate was 91.6%. These findings highlight the feasibility and efficacy of a chemo-free induction and consolidation regimen with ponatinib and blinatumomab in adults with Ph+ ALL, regardless of age. The combination was generally well tolerated, with few treatment discontinuations, even in elderly patients. This suggests that adjusting ponatinib dosing based on age may help reduce severe toxicities (9).

Overall, subcutaneous blinatumomab demonstrated similar or better efficacy than IV blinatumomab with a more convenient administration route. Mini-Hyper-CVD with inotuzumab and blinatumomab improved outcomes, with 1-year OS of 94% vs 51% in control group. Hyper-CVAD with blinatumomab and inotuzumab showed high MRD-negative rates and superior OS, with a 30-month OS of 100% vs 76% in control group. Blinatumomab significantly improved DFS in pediatric ALL, with a 3-year DFS of 96% vs 87.9% in control group, prompting early termination of randomization. Ponatinib + blinatumomab achieved excellent CR and OS rates in Ph+ ALL, outperforming prior ponatinib-based regimens.

## BsAbs immunotherapy in acute myeloid leukemia

AFM28, a bispecific tetravalent innate cell engager (ICE) targeting CD123 and CD16A, was evaluated in a first-in-human phase 1 study among 24 R/R AML patients. AFM28 monotherapy demonstrated early clinical efficacy and a manageable safety profile at doses up to 300 mg per week. In the two highest dose cohorts, 4 out of 12 patients (33.3%) achieved either CR or CRi. At the highest dose (300 mg), 3 out of 6 patients achieved CR or CRi. These findings suggest that AFM28 may hold potential as a treatment for R/R AML (10).

Results from a phase II study of Vibecotamab, a CD123xCD3 bispecific T-cell engager (TCE) antibody, were reported in 37 patients with myelodysplastic syndrome (MDS), chronic myelomonocytic leukemia (CMML) following hypomethylating agent (HMA) treatment failure, and MRD-positive AML. Among the 19 MDS/CMML patients, 13 (68%) responded, with 12 (63%) achieving marrow complete remission (mCR) and 1 (5%) showing hematologic improvement (HI). Of the 16 MDS patients, 9 (56%) achieved mCR, 4 of whom (31%) also showed HI, and 1 (6%) had HI alone. For responders, the median duration of response was 5.2 months, and the overall survival (OS) was 10.3 months. In the 18 AML MRD-positive patients, 5 (28%) achieved MRD negativity, all after just 1 cycle. At the last follow-up, 2 responders relapsed after completing protocol therapy (1.2- and 5.6-months post-treatment), while 3 remained in MRD-negative remission, with durations of 4.1, 24.6, and 25.6 months. This study showed that Vibecotamab was safe and effective for treating low-blast, high-risk myeloid diseases, achieving a 68% response rate in MDS/CMML following HMA treatment failure and a 27% response rate in MRD-positive AML. Notably, 8 of the 10 relapses happened following the protocol therapy completion. As a result, the protocol was amended to allow indefinite treatment with Vibecotamab for responders. The clinical activity of Vibecotamab, particularly in high-risk patients and its lack of significant myelosuppression, suggests it may be a promising candidate for combination therapy in AML, MDS, and CMML (11).

Both studies highlight the potential of targeting CD123 in hematological malignancies, with encouraging results in patient subsets that are typically hard to treat. The therapies could be promising options in relapsed or refractory settings and warrant further investigation, especially in combination strategies.

In summary, the 66th ASH annual meeting showcased encouraging outcomes for various BsAbs in treating R/R ALL and R/R AML, with particularly notable success in R/R ALL trials. These therapies demonstrated significant potential, especially in heavily pretreated patients. Ongoing large-scale studies aim to further assess the efficacy, safety, and toxicity of BsAbs across different treatment settings and in combination with other therapeutic agents. Blinatumomab have received FDA approval for treating relapsed/refractory B-ALL (BLINCYTO^®^ injection for the treatment of adults and children with CD19+B-cell precursor ALLin first or second CR with MRD ≥0.1%, or RR CD19+B-cell precursor ALL). Integrating BsAbs/BITEs into standard treatment regimens has demonstrated improved efficacy, the ability to overcome resistance, and synergy with other immunotherapies. These therapies may also serve as a bridge to CAR-T or hematopoietic stem cell transplantation in relapsed patients. Although challenges such as toxicity—particularly cytokine release syndrome (CRS)—and logistical issues persist, advancements in engineering longer-lasting molecules and combination approaches could help establish them as a cornerstone of cancer treatment.

## Glossary

ALL: acute lymphoblastic leukemia
AML: acute lymphoblastic leukemia
ASH: American Society of Hematology
BsAbs: bispecific antibodies
CHR: complete hematologic remission
CMML: chronic myelomonocytic leukemia
CMR: complete molecular response
CR: complete response
CRh: complete remission with partial hematological recovery
CRi: complete remission with incomplete hematological recovery
D-D: dose-dense
HI: hematologic improvement
HMA: hypomethylating agents
ICE: innate cell engager
INO: Inotuzumab Ozogamicin
mCR: marrow complete remission
MDS: myelodysplastic syndrome
MRD: minimal residual disease
OS: overall survival
RFS: relapse-free survival
R/R: relapsed or refractory
